# Proteomics-based diagnostic peptide discovery for severe fever with thrombocytopenia syndrome virus in patients

**DOI:** 10.1186/s12014-022-09366-w

**Published:** 2022-07-16

**Authors:** Sang-Yeop Lee, Hayoung Lee, Sung Ho Yun, Edmond Changkyun Park, Giwan Seo, Hye-Yeon Kim, Sangmi Jun, Nam Hoon Kim, Dongseob Tark, Ju Yeon Lee, Chang-Seop Lee, Seung Il Kim

**Affiliations:** 1 Research Center for Bioconvergence Analysis, Korea Basic Science Institute, Ochang, 28119 Republic of Korea; 2 Center for Convergent Research of Emerging Virus Infection, Korea Research Institute of Chemical Technology (KRICT), Daejeon, 34114 Republic of Korea; 3 Department of Bio-Analytical Science, University of Science and Technology, Daejeon, 34113 Republic of Korea; 4 Center for Research Equipment, Korea Basic Science Institute, Ochang, 28119 Republic of Korea; 5 Korea Zoonosis Research Institute, Jeonbuk National University, Iksan, Jeonbuk 54531 Republic of Korea; 6 Department of Internal Medicine, Jeonbuk National University Medical School, Jeonju, 54986 Republic of Korea; 7 Biomedical Research Institute of Jeonbuk National University Hospital, Jeonju, 54907 Republic of Korea

**Keywords:** SFTS, Diagnostic, Peptide, Proteomics

## Abstract

**Background:**

Severe fever with thrombocytopenia syndrome (SFTS) virus is an emerging infectious virus which causes severe hemorrhage, thrombocytopenia, and leukopenia, with a high fatality rate. Since there is no approved therapeutics or vaccines for SFTS, early diagnosis is essential to manage this infectious disease.

**Methods:**

Here, we tried to detect SFTS virus in serum samples from SFTS patients by proteomic analysis. Firstly, in order to obtain the reference MS/MS spectral data of SFTS virus, medium from infected Vero cell culture was used for shotgun proteomic analysis. Then, tryptic peptides in sera from SFTS patients were confirmed by comparative analysis with the reference MS/MS spectral data of SFTS virus.

**Results:**

Proteomic analysis of culture medium successfully discovered tryptic peptides from all the five antigen proteins of SFTS virus. The comparative spectral analysis of sera of SFTS patients revealed that the N-terminal tryptic peptide of the nucleocapsid (N) protein is the major epitope of SFTS virus detected in the patient samples. The prevalence of the peptides was strongly correlated with the viral load in the clinical samples.

**Conclusions:**

Proteomic analysis of SFTS patient samples revealed that nucleocapsid (N) protein is the major antigen proteins in sera of SFTS patients and N-terminal tryptic peptide of the N protein might be a useful proteomic target for direct detection of SFTS virus. These findings suggest that proteomic analysis could be an alternative tool for detection of pathogens in clinical samples and diagnosis of infectious diseases.

**Supplementary Information:**

The online version contains supplementary material available at 10.1186/s12014-022-09366-w.

## Introduction

Severe fever with thrombocytopenia syndrome (SFTS) virus (SFTSV) is a causative agent of SFTS, which is a new emerging infectious disease with no approved therapeutic or vaccines and has high mortality rate (more than 30%) [1]. The major clinical features of SFTS are myalgia, high fever, fatigue, abdominal pain, and nausea/vomiting [2, 3]. The SFTS virus has a three segmented genome: the L segment encodes the RNA-dependent RNA polymerase (RdRP), the M segment encodes glycoproteins (Gn and Gc), and the S segment encodes the nucleocapsid (N) and nonstructural proteins (NS). Gn and Gc form a heterodimer on the surface of the virus, and the N proteins function as a scaffold to facilitate packing of virus particles.

Early diagnosis is essential to manage SFTS since the lethality of SFTS is relatively high and the clinical manifestations are non-specific [4]. Several methods are used to diagnose SFTS. In general, molecular diagnostic approaches are preferred due to their high sensitivity and selectivity [5]. Real-time RT-PCR and RT-LAMP tests are developed to detect SFTSV directly [6–9]. Serological tests are also important diagnostic tools; these tests detect immunoglobulins (IgM and IgG) that target antigenic SFTSV proteins in human serum [10, 11]. Recently, monoclonal antibodies specific for the N protein of SFTSV were developed for use in SFTS antigen detection tests [3, 12]. Additionally, direct observation of SFTSV by electron microscopy was reported as an alternative diagnostic method [13, 14]. However, direct detection of SFTSV in patients by targeted-proteomic was not reported yet.

In general, direct detection of pathogenic viruses in patients using proteomic has not been frequently reported. This is due mainly to technical and clinical difficulties to overcome limit of detection (LOD) because concentration of pathogenic viruses is very low and duration of detectible virus in the host cells is very short. In addition, abundant host cell proteins increase the complexity of clinical samples and hinder specific detection of target viruses. However, direct detection of pathogenic virus is important for understanding the mechanism of infection and for screening of diagnostic antigens. Therefore, improved novel proteomic approaches for direct detection of pathogenic virus are required [15, 16]. The information provided by proteomic-based approaches will be valuable for planning strategies regarding selection of target proteins and generation of diagnostic antibodies specific for target peptides or proteins. Recently, many researchers have been paying close attention to methods that enable direct detection of SARS-CoV-2; indeed, SARS-CoV-2 proteins can now be detected in gargle solutions, nasal swabs, and scrapings of the epithelium of COVID-19 patients [17–20].

Here, we performed proteomic analysis of serum specimens from SFTS patients to detect SFTS virus directly. For this purpose, we used proteomic data derived from analysis of virus culture medium as a reference for comparative spectral analyses. To the best of our knowledge, this is the first report describing a proteomic assay for direct detection of SFTSV in patient serum.

## Methods

### Sample preparation of culture cell and patient’s sera

African green monkey kidney cell line (Vero E6 ATCC CRL-1586) was used for the amplification of SFTSV. Cells were cultured in complete media (DMEM with 10% Fetal bovine serum, 1× Penicillin–Streptomycin media) at 37 ℃ with 5% CO_2_. Human Origin SFTSV (KADGH; NCCP43261) was donated from KCDC. SFTS virus was inoculated into monolayer of Vero E6 cell, which was cultured in inoculate culture media (DMEM with 1× penicillin–Streptomycin) for 60 min. Culture dish was mildly shaken at 15 min intervals to increase efficiency of inoculation. Inoculated cells were transferred into new culture media (DMEM with 2% FBS, 1× penicillin–streptomycin) and cultured for 5 days. Supernatant was centrifuged at 15,000 rpm (20,000*g*) for 10 min at room temperature and precipitates were used for next step of sample preparation. Sera of patients also treated as same procedure. Samples (precipitates of cultured cells and sera) were mixed with same volume of lysate buffer (25 mM ammonium bicarbonate, 4% sodium dodecyl sulfate) and boiled for 10 min. Supernatant of reaction solution was prepared by centrifugation at 15,000 rpm (20,000*g*) for 10 min. As final step of sample preparation, albumin depletion kit (85160, Thermo Scientific, USA) was used for albumin removal and each sample was used for proteomic analysis of SFTS virus and cultured cell.

### Proteomic analysis and bioinformatic analysis

Prepared sample proteins were separated by 12.5% SDS-PAGE and performed in-gel tryptic digestion by previously reported methods [21]. MS/MS analysis was performed using Q Exactive Plus mass spectrometer (Thermo Scientific, USA). MS/MS data of cultured SFTS virus were analyzed by using MASCOT 2.4 with an integrated database that was constructed with Uniprot Human proteome database and integrated SFTS virus database. The integrated SFTSV database was constructed by combining the protein sequences which were downloaded from the ViPR (https://www.viprbrc.org/) with SFTS virus KACNH3 (Accession No. of NCBI KP663743–KP663745), isolated from Korea, as a reference. In general, proteomic detection of pathogens such as viruses or bacteria in patient serum has serval technical hurdles to overcome. First, pathogens are present at very low concentrations in specimens of patients. Second, the presence of serum abundant proteins (such as albumins, globulins, and fibrinogen), can be a hindrance to detect low copy number of proteins originated from pathogens. Last, but not least, is the lack of a suitable proteomics database and/or informatics tools for precise identification of pathogenic viruses or bacteria. To minimize these difficulties, we used patient serum samples containing different concentrations of virus to identify the optimal virus concentration for clinical proteomics. We also used an albumin depletion kit to reduce the complexity of samples, as well as spectral analysis programs and an in-house database of SFTS viruses. Finally, we used MS/MS spectral data derived from analysis of SFTSV cultured in Vero E6 cells as a reference; this enabled us to obtain accurate spectra data for SFTSV in clinical samples. The peptides of SFTS virus from patients were identified by COSS 1.0, using spectral library of SFTSV cultured in Vero E6 cells [22]. The spectral library for COSS analysis was constructed using the results of PeptideProphet and Spectra ST of the Trans-Proteomic Pipeline (TPP) [23]. The identified spectral peaks of SFTS virus proteins were confirmed by PEAKS Studio 7.0 (Bioinformatics Solution Inc., ON, Canada).

### LC-parallel reaction monitoring (PRM) MS/MS analysis

Additional peptide identification was performed using the same instrument in PRM mode. First, 11 peptides of the NP protein were selected as targets (based on uniqueness) for the PRM method. Two target peptides (ELAYEGLDPALIIK, aa 27–40; and GILGPDGVPSR, aa 223–233) were finally selected for LC-PRM MS/MS analysis based on the spectral count, length, hydropathy, reactive residues, and modification motifs, as previously discussed [24]. Their stable isotope-labeled peptides (heavy peptides, Lysine-13C(6)15 N(2) and Arginine-13C(6)15 N(4)) were purchased from AnyGen (Gwangju, Korea) to develop the PRM assay. Second, synthetic peptides (1 pmol/μL each) were analyzed to optimize parameters. Target peptides and a list of transitions were selected from Skyline platform version 21.2 (MacCoss Lab Software; https://skyline.ms). These results were also used to generate spectral libraries. The standard samples were separated with 0.1% formic acid in water and acetonitrile/0.1% formic acid, using a 35 min gradient at a flow rate of 250 nL/min. The maximum acquisition time and automatic gain control were set at 100 ms and 5 × 10^4^, respectively. Thirdly, the standard method was applied for correct peptide identification, as heavy peptides co-elute with the peptides of interest. A blank solvent (0.1% formic acid in water) was injected between samples to prevent sample carryover. The mProphet method was used for peak picking, FDR estimation with a reverse database (filtered using a q-value < 0.01), and data validation [25]. The peak area ratio to heavy peak areas was used for quantification.

### Structural analysis of tryptic peptides in overall structure of SFTSV N protein

The 3D structures of SFTSV N protein have been previously reported by Zhou et al. and Jiao et al. [26, 27]. We analyzed where the sequences of the patient-derived SFTSV peptides correspond to in the overall structure of the full-length N protein (PDB code 4J4R). The molecular model figure was generated using PyMol program.

## Results

### Proteomic analysis of culture medium from Vero E6 cells infected with SFTSV

**Fig. 1:**
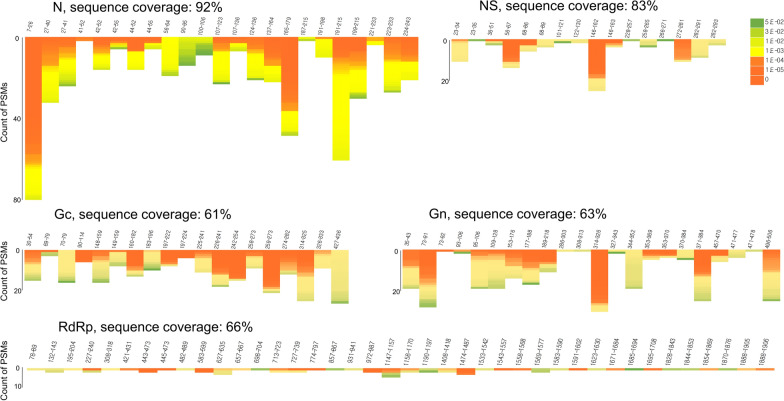
Proteomic analysis of severe fever with thrombocytopenia syndrome (SFTS) virus cultured in Vero E6 cells. The N, Gc, Gn, L, and NS proteins of SFTS virus were identified. The Sequence coverage (%) and detection frequency of each protein (Peptide spectral match) are indicated. MS/MS analysis identified nearly all tryptic peptides from the N protein (> 92% sequence coverage); however, detection frequency was highest for the N-terminal tryptic peptide (amino acids 7–26) of the N protein

The initial step of proteomic identification of SFTSV was to use culture medium from infected Vero E6 cells as a reference to obtain spectral data. Precipitates of culture medium were subjected to tryptic digestion prior to shotgun proteomic analysis using a Q Exactive Plus mass spectrometer. Sequence coverage of each protein components of SFTS virus was in the range of 61–92%. In particular, the sequence coverage of the N protein was high (92%) (Fig. 1). Considering that each protein component has a unique copy number in SFTS virus, this semi-quantitative proteomic data reveals the abundancy of each protein, as well as which proteins are likely to be the best diagnostic makers. Based on our proteomic results, we suggest the N proteins can be strong candidates for SFTS diagnosis. An interesting point is that each tryptic peptide derived from the N protein showed a different peptide-spectrum match (PSM). In particular, the N-terminal tryptic peptide (7–26th) had the highest score. Next, we selected six SFTS patients and used their sera for proteomic analysis. The clinical characteristics of the six patients and virus CT values are summarized in Table 1 and Additional file 1: Table S1, respectively. The estimated virus copy number for each patient ranged from 2.18 × 10^4^ to 3.03 × 10^1^ copies/mL, based on Ct values for the M segment. Each tryptic peptide derived from SFTSV proteins detected using the proteomics method is summarized in Table 2. Identified peptides were confirmed by comparative spectral analysis of culture cell medium and patient serum (Additional file 2: Fig. S1). The results showed that tryptic peptides derived from SFTSV were detected mainly in two SFTS patients (SFTS-007 and SFTS-024) with a high viral load. However, few if any tryptic peptides were detected in the other four patients (SFTS-032, SFTS-033, SFTS-034, and SFTS-041). Among the identified tryptic peptides, the N-terminal tryptic peptide (7–26th) derived from the N protein was detected most frequently (Table 2).

**Table 1:** Demographic, clinical characteristics and laboratory findings of SFTS patients

Characteristics	SFTS (n = 6)
Epidemiology, no. (%)	
Age, mean Y ± SD (range)	74.33 ± 10.97
Female	4 (66.7)
Occupational exposure	2 (33.3)
Comorbidities, no. (%)	
Cardiovascular disease^a^	0
Cerebrovascular disease	0
Pulmonary disease^b^	0
Chronic kidney disease	0
Diabetes mellitus	1 (16.7)
Malignancies	0
Clinical signs and symptoms, no. (%)	
Headache	2 (33.3)
Dyspepsia	3 (50.0)
Nausea/vomiting	3 (50.0)
Abdominal pain	3 (50.0)
Chills	5 (83.3)
Myalgia	5 (83.3)
Fatigue	5 (83.3)
Rash/eschar	3 (50.0)
Tick bite	3 (50.0)
Laboratory values, median (IQR)	
WBC count, ×1000/mm^3^	1.6 (1.2–2.4)
Platelet count, ×1000/mm^3^	100.0 (51.0–118.0)
aPTT, sec	34.7 (27.8–44.0)
Total bilirubin, mg/dL	0.35 (0.25–0.95)
Albumin, g/dL	4.0 (3.6–4.7)
AST, IU/L	148.5 (26.0–727.0)
ALT, IU/L	104.0 (15.0–190.0)
LD, IU/L	673.5 (421.0–1914.0)
Creatinine, mg/dL	0.7 (0.5–1.3)
hs-CRP, mg/dL	4.5 (0.2–13.7)
Clinical outcomes	
Length of hospital stay, median (IQR)	9 (7.0–18.0)
In-hospital mortality, no. (%)	2 (33.3)

SFTS, severe fever with thrombocytopenia syndrome; SD, standard deviation; IQR, interquartile range; WBC, white blood cell; aPTT, activated partial thromboplastin time; AST, aspartate aminotransferase; ALT, alanine aminotransferase; LD, lactate dehydrogenase; CRP, C-reactive protein

^a^Includes myocardial infarction, congestive heart failure, and peripheral vascular disease

^b^Chronic obstructive pulmonary disease, asthma

**Table 2:** Proteomic detection of tryptic peptides of SFTS virus prepared from the serum of SFTS patients

Accession No.	Gene	Peptides (amino acid residues)	SFTS patients								
			SFTS-007			SFTS-024			SFTS-032		
AJO16100.1	NP	7–26	5^a^	5^b^	5^c^	2	3	3	–	–	–
	NP	27–40	1	3	3	1	1	1	–	–	–
	NP	56–64	–	1	1	–	1	–	–	–	–
	NP	90–95	–	1	1	–	1	1	–	–	–
	NP	96–106	–	1	1	–	1	1	–	–	–
	NP	100–106	1	1	1	1	1	1	–	–	–
	NP	107–123	–	–	–	–	–	–	–	–	–
	NP	124–136	1	1	1	1	1	–	–	–	–
	NP	165–179	1	–	–	–	–	–	–	–	–
	NP	191–215	2	2	2	–	–	–	–	–	–
	NP	223–233	2	2	2	2	2	2	–	–	–
	NP	234–243	–	1	1	–	1	1	–	–	–
AJO16097.1	RdRp	94–117	–	–	–	–	1	1	–	–	–
	RdRp	156–164	–	–	–	–	1	1	–	–	1
	RdRp	207–226	–	–	–	–	–	–	1	–	–
	RdRp	297–307	–	–	–	–	–	–	–	–	1
	RdRp	322–331	–	–	1	–	4	4	–	–	–
	RdRp	399–405	–	–	1	–	–	–	–	–	–
	RdRp	421–432	–	–	–	–	–	–	–	–	–
	RdRp	482–507	–	–	2	–	2	–	–	–	–
	RdRp	690–695	–	–	–	–	–	–	–	–	–
	RdRp	836–849	–	–	2	–	1	1	–	–	–
	RdRp	1431–1440	–	–	2	–	3	3	–	–	–
	RdRp	1441–1451	–	–	–	–	1	1	–	–	–
	RdRp	1543–1557	1	–	–	–	–	–	–	–	–
	RdRp	1828–1843	1	–	–	–	–	–	–	–	–
	RdRp	1854–1869	1	–	–	–	–	–	–	–	–
	RdRp	1877–1887	–	–	1	–	–	–	–	–	–
AJO16099.1	NS	23–34	–	–	–	–	–	–	2	–	–
	NS	36–51	3	–	–	–	–	–	–	–	–
	NS	68–86	1	–	–	1	–	–	1	–	–
	NS	68–89	1	–	–	1	–	–	1	–	–
	NS	282–293	–	–	–	–	–	–	–	–	1
AJO16098_1_1705_3237.1	GC	160–182	–	–	–	1	–	–	–	–	–
	GC	197–222	–	–	–	2	–	–	–	–	–
	GC	225–241	–	–	–	–	–	–	1	–	–
	GC	258–273	–	–	–	–	–	–	1	–	–
	GC	274–282	1	–	–	–	–	–	–	–	–
	GC	314–325	2	–	–	–	–	–	–	–	–
AJO16098_1_76_1623.1	GN	109–128	–	–	–	1	–	–	–	–	–
	GN	314–326	–	–	1	–	–	1	–	–	–
	GN	371–384	1	–	–	–	–	–	–	–	–

Accession No.	Gene	Peptides (amino acid residues)	SFTS patients								
			SFTS-033			SFTS-034			SFTS-041		
AJO16100.1	NP	7–26	–	–	–	–	–	–	–	–	–
	NP	27–40	1	–	–	–	–	–	–	–	–
	NP	56–64	–	–	–	–	–	–	–	–	–
	NP	90–95	–	–	–	–	–	–	–	–	–
	NP	96–106	–	–	2	–	–	–	–	–	1
	NP	100–106	–	–	–	–	–	–	–	–	–
	NP	107–123	–	–	–	1	–	–	–	–	–
	NP	124–136	–	–	–	–	–	–	–	–	–
	NP	165–179	–	–	–	–	–	–	–	–	–
	NP	191–215	–	–	–	–	–	–	–	–	–
	NP	223–233	–	–	–	–	–	–	–	–	–
	NP	234–243	–	–	–	–	–	–	–	–	–
AJO16097.1	RdRp	94–117	–	–	–	–	–	–	–	–	–
	RdRp	156–164	–	–	1	–	–	–	–	–	–
	RdRp	207–226	–	–	–	–	–	–	–	–	–
	RdRp	297–307	–	–	–	–	–	–	–	–	–
	RdRp	322–331	–	–	–	–	–	–	–	–	–
	RdRp	399–405	–	–	–	–	–	–	–	–	–
	RdRp	421–432	–	–	–	–	–	1	–	–	–
	RdRp	482–507	–	–	–	–	–	–	–	–	–
	RdRp	690–695	–	–	1	–	–	–	–	–	–
	RdRp	836–849	–	–	–	–	–	–	–	–	–
	RdRp	1431–1440	–	–	–	–	–	–	–	–	–
	RdRp	1441–1451	–	–	–	–	–	–	–	–	–
	RdRp	1543–1557	–	–	–	–	–	–	–	–	–
	RdRp	1828–1843	–	–	–	–	–	–	–	–	–
	RdRp	1854–1869	–	–	–	–	–	–	–	–	–
	RdRp	1877–1887	–	–	–	–	–	–	–	–	–
AJO16099.1	NS	23–34	–	–	–	–	–	–	–	–	–
	NS	36–51	–	–	–	1	–	–	–	–	–
	NS	68–86	–	–	–	–	–	–	–	–	–
	NS	68–89	–	–	–	–	–	–	–	–	–
	NS	282–293	–	–	–	–	–	–	–	–	–
AJO16098_1_1705_3237.1	GC	160–182	1	–	–	–	–	–	–	–	–
	GC	197–222	–	–	–	–	–	–	–	–	–
	GC	225–241	–	–	–	–	–	–	–	–	–
	GC	258–273	–	–	–	–	–	–	–	–	–
	GC	274–282	–	–	–	–	–	–	–	–	–
	GC	314–325	–	–	–	–	–	–	–	–	–
AJO16098_1_76_1623.1	GN	109–128	–	–	–	–	–	–	–	–	–
	GN	314–326	–	–	–	–	–	–	–	–	–
	GN	371–384	–	–	–	–	–	–	–	–	–

^a^Tryptic peptides were identified using COSS

^b^Tryptic peptides were identified using Mascot (v2.4) with SFTS virus DB

^c^Tryptic peptides were identified using Mascot (v2.4) with integrated DB of Human and SFTS virus

### Validation of SFTS NP in serum using LC-PRM MS/MS

**Fig. 2:**
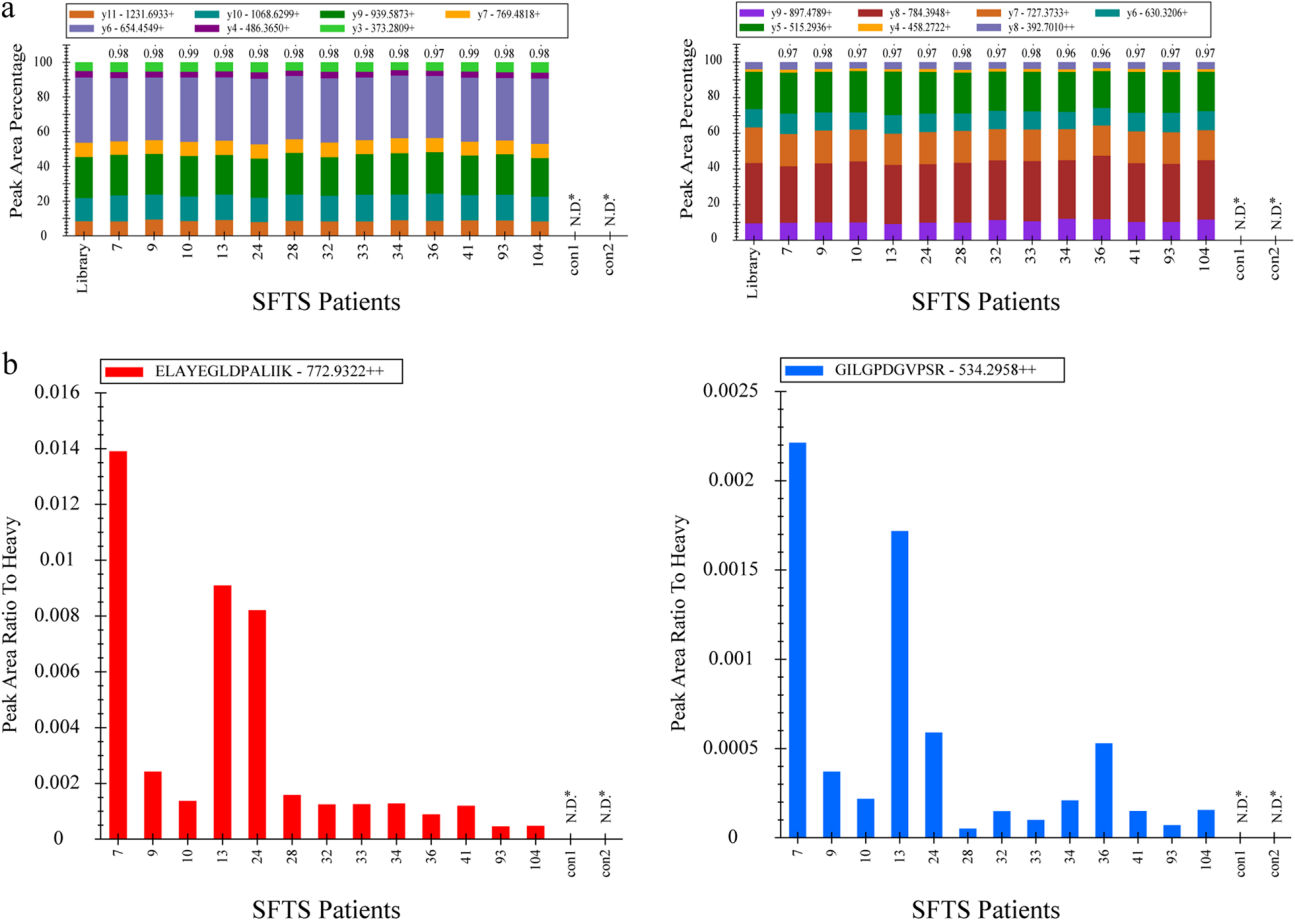
Qualitative characteristics of the PRM assay. **a** All PRM transitions for target peptides were compared with their corresponding spectral library transitions. Each color bar represents one transition ion and its relative intensity among the others. The dot product (dotp) annotated above the bar graph is the normalized dot product of the light transition peak areas with the corresponding intensity in the library. **b** Relative quantification of target peptides in sera specimens of 13 SFTS patients and two normal subjects. The values were calculated based on each heavy peptide (1 pmol) peak area. The red bar denoted ELAYEGLDPALIIK peptides and blue bar denoted GILGPDGVPSR peptides. N.D.*Not detected

The previously identified peptides of NP protein were validated by PRM-MS analysis in thirteen SFTS patients and two normal subjects, including six SFTS patients analyzed in shotgun proteomics. Among the NP protein-derived peptides identified, we selected two proteotypic peptides (ELAYEGLDPALIIK and GILGPDGVPSR) for further investigation. Details about each peptide are provided in Additional file 3: Table S2. We estimated the concentration of targets in patient serum using stable isotope-labeled peptides (each at 1 pmol/μL). The chromatogram, retention times, and transition rank order of the detected peptides matched well with those of the heavy peptides (Additional file 4: Fig S2). Also, all dot product (dotp) values were 0.96 or higher, suggesting that the identified peptides were derived from SFTS virus in serum (Fig. 2a). Thus, the two peptides derived from the N protein were identified in all 13 SFTS patients. The peak area ratio to heavy peptide is shown in Fig. 2b. Patient numbers 7, 13, and 24 show higher values than the others, which corresponds with the previous results.

## Discussion

Here, we performed proteomic analysis of serum from SFTS patients. Culture medium from Vero cells infected with SFTSV was used as a reference for comparative analysis of mass spectra. Serum samples from six SFTS patients were used to identify pathogen-derived tryptic peptides. We found that the N-terminal tryptic peptide (7–26th) of the N protein was the major SFTSV-derived peptide detected in serum samples from two patients (SFTS-007 and SFTS-024). Analysis of these two patient samples suggested that the LOD of label-free LC–MS/MS shotgun proteomics ranges from 10^2^–10^3^ copies/mL. However, the results suggest that consistent and reliable detection of SFTSV using Label-free LC–MS/MS shotgun proteomics requires at least 10^4^ copies/mL. Thus, we performed LC–PRM MS/MS to validate N proteins in patients with SFTS. We found that two tryptic peptides (ELAYEGLDPALIIK, aa 27–40; and GILGPDGVPSR, aa 223–233) were present in all 13 SFTS patients and two normal subjects. Although the shotgun proteomics method is less sensitive than PRM MS/MS, the peptides identified by the shotgun method were consistent with those identified by PRM MS/MS. It seems that our identifying approaches to finding pathogen-derived peptides were suitable in Label-free LC–MS/MS shotgun proteomics. However, quantitative results showed inconsistency with respect to Ct values and detection rates of SFTS viral peptides in SFTS patients. Therefore, far more patient samples will be needed to obtain more accurate data regarding the LOD of MS/MS for SFTSV. The proteomics data presented herein raise the question of why tryptic peptides (particularly the N-terminal tryptic peptide) of the N protein are detected more easily than those of other SFTSV proteins. The most likely explanation is the 3D structure of the N-protein. We elucidated the predicted 3D structure of the N-protein using the PyMol program (Additional file 5: Fig. S3). The results suggest that the N-terminal region, which comprises two tryptic peptides (7–26th and 27–40th) of the N protein is exposed on the outside of the structure, making it more prone to denaturation and tryptic digestion. Thus, these two tryptic peptides would be more detectible by MS/MS analysis. In addition, the N-terminal region of the N protein may be highly immunogenic because host immune system. Yu et al. used phage library approach to generate monoclonal antibodies (mAbs) specific for the N protein of SFTSV virus; they found that the N-terminal region of the N protein was the major binding site for most of the mAbs generated [12]. This result supports our assumption that the N-terminal region of SFTSV is more immunogenic and important binding sites with mAbs against N-proteins. Therefore, mAbs specific for the N-terminal region of the N protein will be useful in lateral flow tests or antibody/antigen based-biosensors for detection of SFTSV. In general, the LOD of lateral flow tests and antibody/antigen-based biosensors is around 10^2^–10^6^ copies/mL according to the performance of antibody-antigen reaction [28, 29]. Therefore, the data presented herein suggest that sensitive proteomics analysis approaches are an alternative tool for detection and diagnosis of SFTSV in clinical samples. Additionally, the results suggest that LC-based proteomics analysis is a useful tool for screening diagnostic peptides-derived from pathogenic viruses.

## Conclusions

Proteomic analysis of SFTS patient samples revealed that nucleocapsid (N) protein is the major antigen proteins in sera of SFTS patients and N-terminal tryptic peptide of the N protein might be a useful proteomic target for direct detection of SFTS virus. These findings suggest that proteomic analysis could be an alternative tool for detection of pathogens in clinical samples and diagnosis of infectious diseases.

## Supplementary Information

**Additional file 1:** **Table S1. **Results of molecular diagnostic using RT-PCR of SFTS patients.

**Additional file 2**: **Figure S1.** MS/MS spectra of tryptic peptides derived from the N protein in serum samples from patients infected with severe fever with thrombocytopenia syndrome virus (SFTSV). MS/MS spectra for each peptide in medium from cultured cells or in serum from SFTS patients were compared. A: IAVEFGEQQLNLTELEDFAR (7–26th); B: ELAYEGLDPALIIK (27–40th); C: LSITPVR (100–106th); D: GILGPDGVPSR (223–233th).

**Additional file 3:** **Table S2. **Target peptides and transitions used for the LC-PRM MS Analysis.

**Additional file 4**: **Figure S2.** Qualitative characteristics of the PRM assay. The representative chromatograms of target peptides used in the PRM assay. a) Upper rows designated native peptides in serum specimens and b) lower rows designated heavy peptides of them. Mass error and retention times are annotated on the peak. c) Retention time of two target native peptides (light) and their synthetic peptides (heavy). The red bar denoted light peptides and blue bar denoted heavy peptides.

**Additional file 5: Figure S3.** 3D structure of the N protein and peptides detected by MS/MS analysis. Each peptide is assigned a different color: red (7–26th), orange (100–136th), yellow (165–179th), green (191–215th), or blue (223–233th).

## Data Availability

All data generated during this study are included in this published article. Total list of identified proteins has been uploaded as additional files.

## Abbreviations

SFTS: Severe fever with thrombocytopenia syndrome
SFTSV: Severe fever with thrombocytopenia syndrome virus
N: Nucleocapsid
RdRP: RNA-dependent RNA polymerase
Gn: Glycoprotein N
Gc: Glycoprotein C
NS: Nonstructural proteins
LOD: Limit of detection
PSM: Peptide-spectrum match
mAbs: Monoclonal antibodies
