# Supplementary material for: Proteomics-based diagnostic peptide discovery for severe fever with thrombocytopenia syndrome virus in patients
Source: Clin Proteomics. 2022 Jul 16;19:28. doi: 10.1186/s12014-022-09366-w (PMC9287713; doi:10.1186/s12014-022-09366-w)
Supplement: Supplementary file 1 — Additional file 1: Table S1. Results of molecular diagnostic using RT-PCR of SFTS patients. [file 12014_2022_9366_MOESM1_ESM.docx]

**Additional File 1: Table S1. Results of molecular diagnostic using RT-PCR of SFTS patients**

|  |  |  | Real-time RT-PCR *C*_t_ value | | Copies no. (copy/ml)* | |
| --- | --- | --- | --- | --- | --- | --- |
| Patient ID | Sex | Age | S segment | M segment | S segment | M segment |
| SFTS-07 | F | 82 | 23.68 | 25.14 | 4.81 x10^4^ | 2.18 x10^4^ |
| SFTS-24 | F | 64 | 31.50 | 29.20 | 1.60 x10^2^ | 2.90 x10^3^ |
| SFTS-32 | F | 60 | 28.48 | 26.29 | 1.50 x10^3^ | 8.95 x10^3^ |
| SFTS-33 | F | 78 | 31.19 | 32.19 | 2.12 x10^2^ | 9.37 x10^1^ |
| SFTS-34 | M | 73 | 31.01 | 32.39 | 2.42 x10^2^ | 8.03 x10^1^ |
| SFTS-41 | M | 89 | 31.90 | 33.65 | 1.27 x10^2^ | 3.03 x10^1^ |
| SFTS-09 | M | 75 | 36.88 | 32.64 | 3.49 x10^1^ | 6.62 x10^1^ |
| SFTS-10 | F | 90 | 31.87 | 29.23 | 1.30 x10^2^ | 9.23 x10^2^ |
| SFTS-13 | M | 62 | 21.16 | 22.26 | 2.96 x10^5^ | 2.01 x10^5^ |
| SFTS-28 | F | 82 | 27.43 | 25.08 | 3.21 x10^3^ | 2.28 x10^4^ |
| SFTS-36 | M | 50 | 32.91 | 30.80 | 6.14 x10^1^ | 2.74 x10^2^ |
| SFTS-104 | F | 78 | NA | NA | NA | NA |
| SFTS-93 | F | 64 | 23.56 | 22.26 | 5.24 x10^4^ | 2.01 x10^5^ |

*The copy number per milliliter of SFTS virus in serum of patient was calculated according to standard curve [6].
