# Supplementary material for: Proteomics-based diagnostic peptide discovery for severe fever with thrombocytopenia syndrome virus in patients
Source: Clin Proteomics. 2022 Jul 16;19:28. doi: 10.1186/s12014-022-09366-w (PMC9287713; doi:10.1186/s12014-022-09366-w)
Supplement: Supplementary file 2 — Additional file 2: Figure S1. MS/MS spectra of tryptic peptides derived from the N protein in serum samples from patients infected with severe fever with thrombocytopenia syndrome virus (SFTSV). MS/MS spectra for each peptide in medium from cultured cells or in serum from SFTS patients were compared. A: IAVEFGEQQLNLTELEDFAR (7–26th); B: ELAYEGLDPALIIK (27–40th); C: LSITPVR (100–106th); D: GILGPDGVPSR (223–233th). [file 12014_2022_9366_MOESM2_ESM.pptx]

## Slide 1
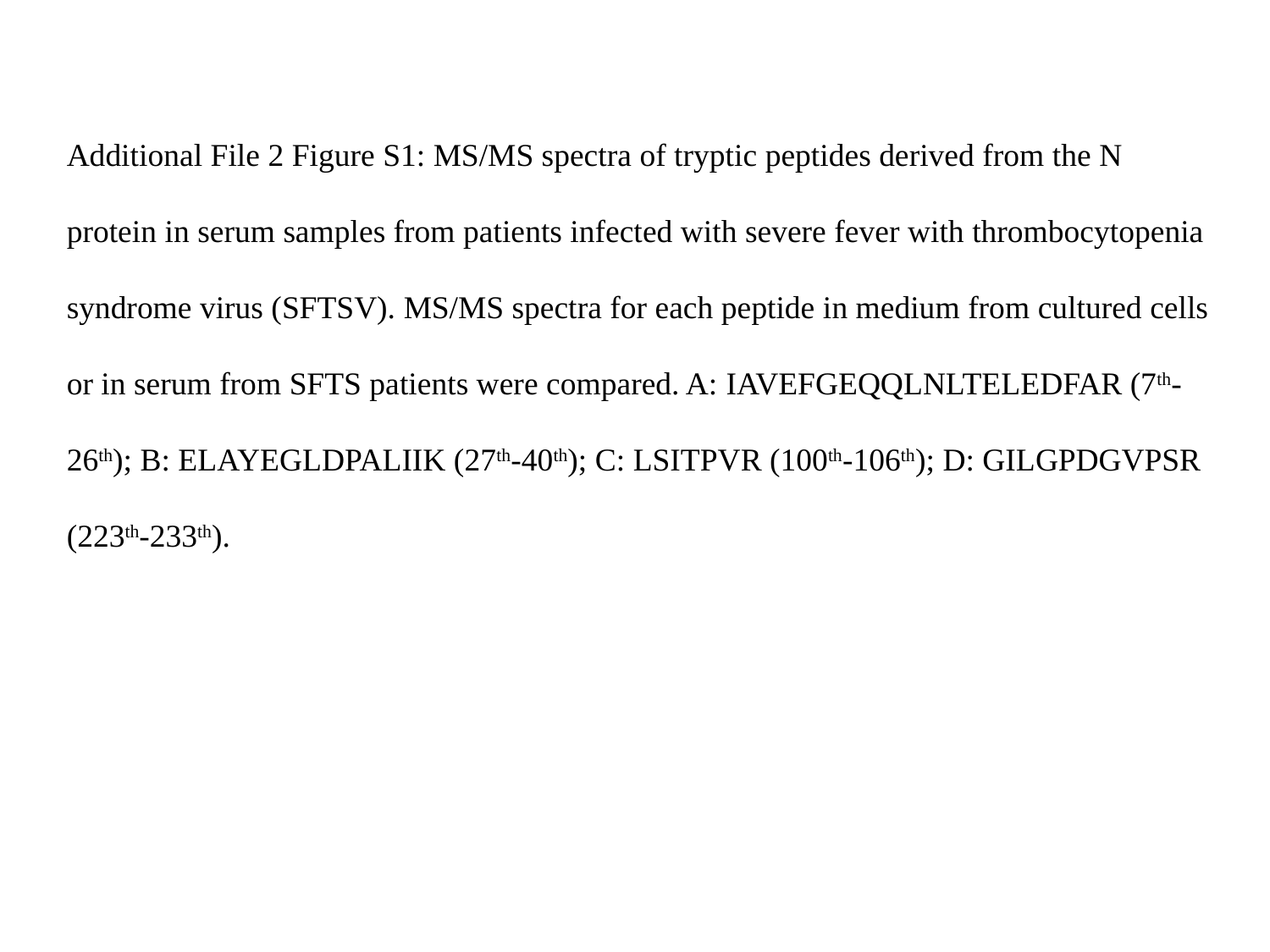

Additional File 2 Figure S1: MS/MS spectra of tryptic peptides derived from the N protein in serum samples from patients infected with severe fever with thrombocytopenia syndrome virus (SFTSV). MS/MS spectra for each peptide in medium from cultured cells or in serum from SFTS patients were compared. A: IAVEFGEQQLNLTELEDFAR (7th-26th); B: ELAYEGLDPALIIK (27th-40th); C: LSITPVR (100th-106th); D: GILGPDGVPSR (223th-233th).

## Slide 2
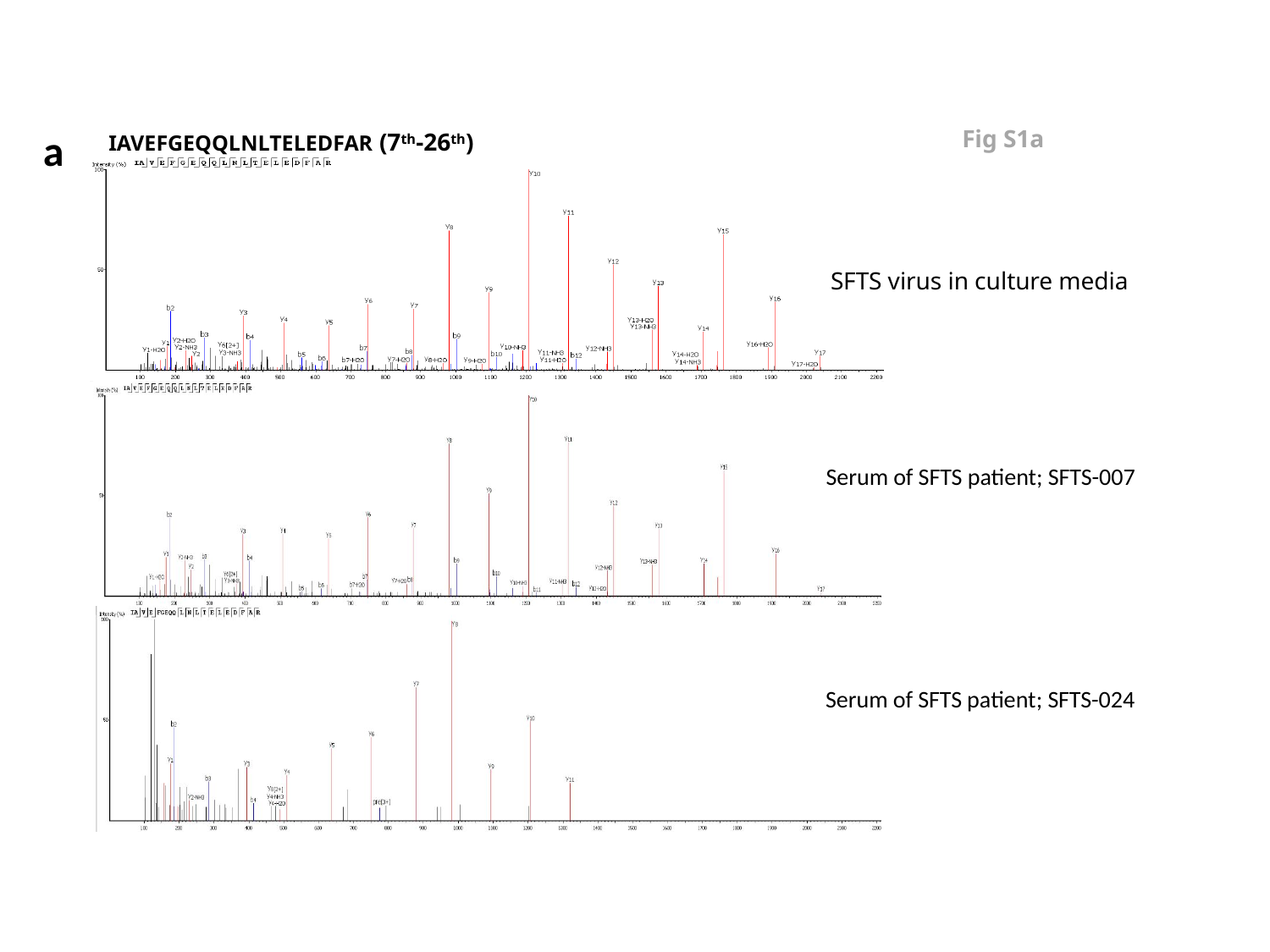

a
Fig S1a
IAVEFGEQQLNLTELEDFAR (7th-26th)
SFTS virus in culture media
Serum of SFTS patient; SFTS-007
Serum of SFTS patient; SFTS-024

## Slide 3
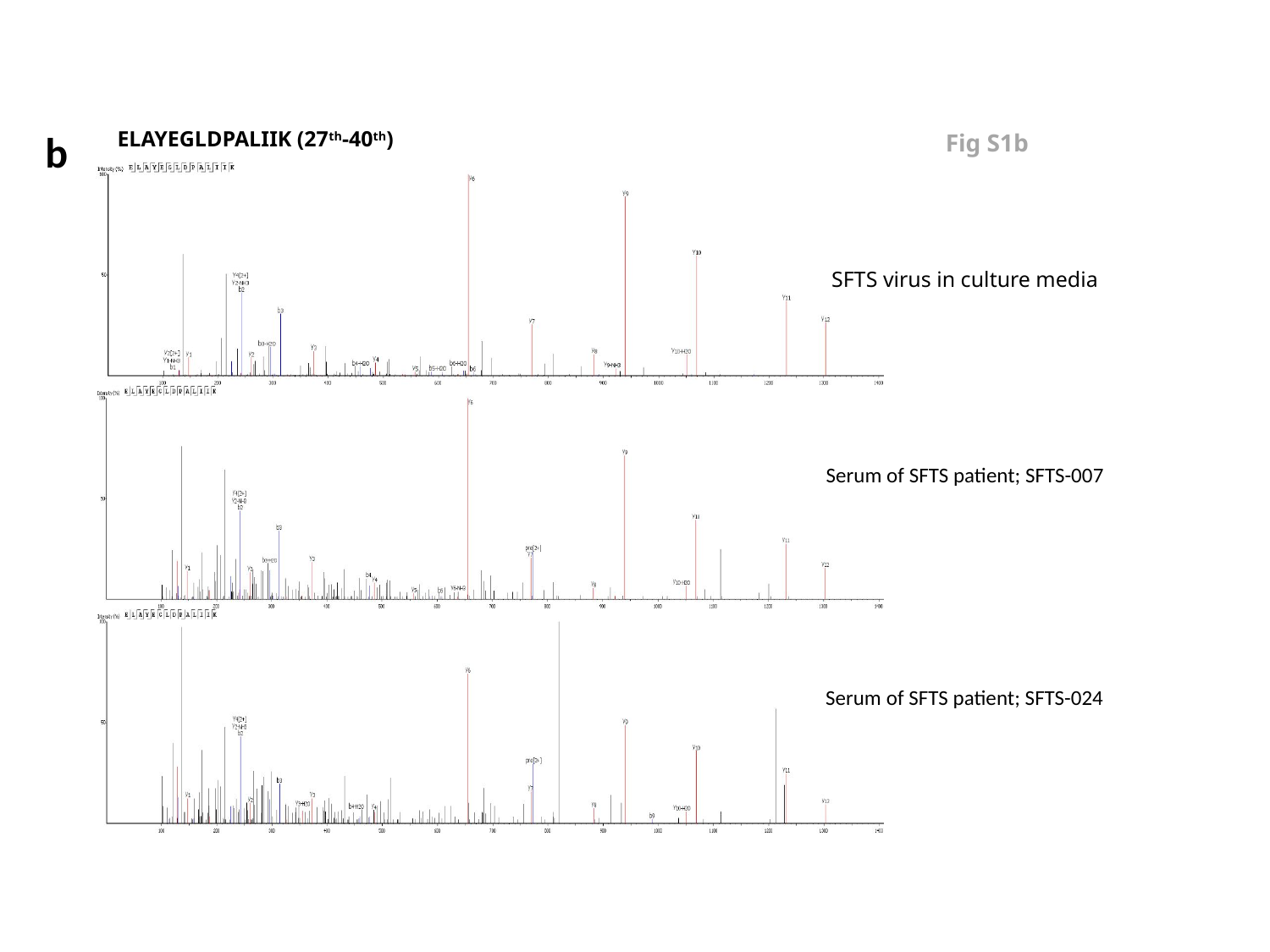

b
ELAYEGLDPALIIK (27th-40th)
Fig S1b
SFTS virus in culture media
Serum of SFTS patient; SFTS-007
Serum of SFTS patient; SFTS-024

## Slide 4
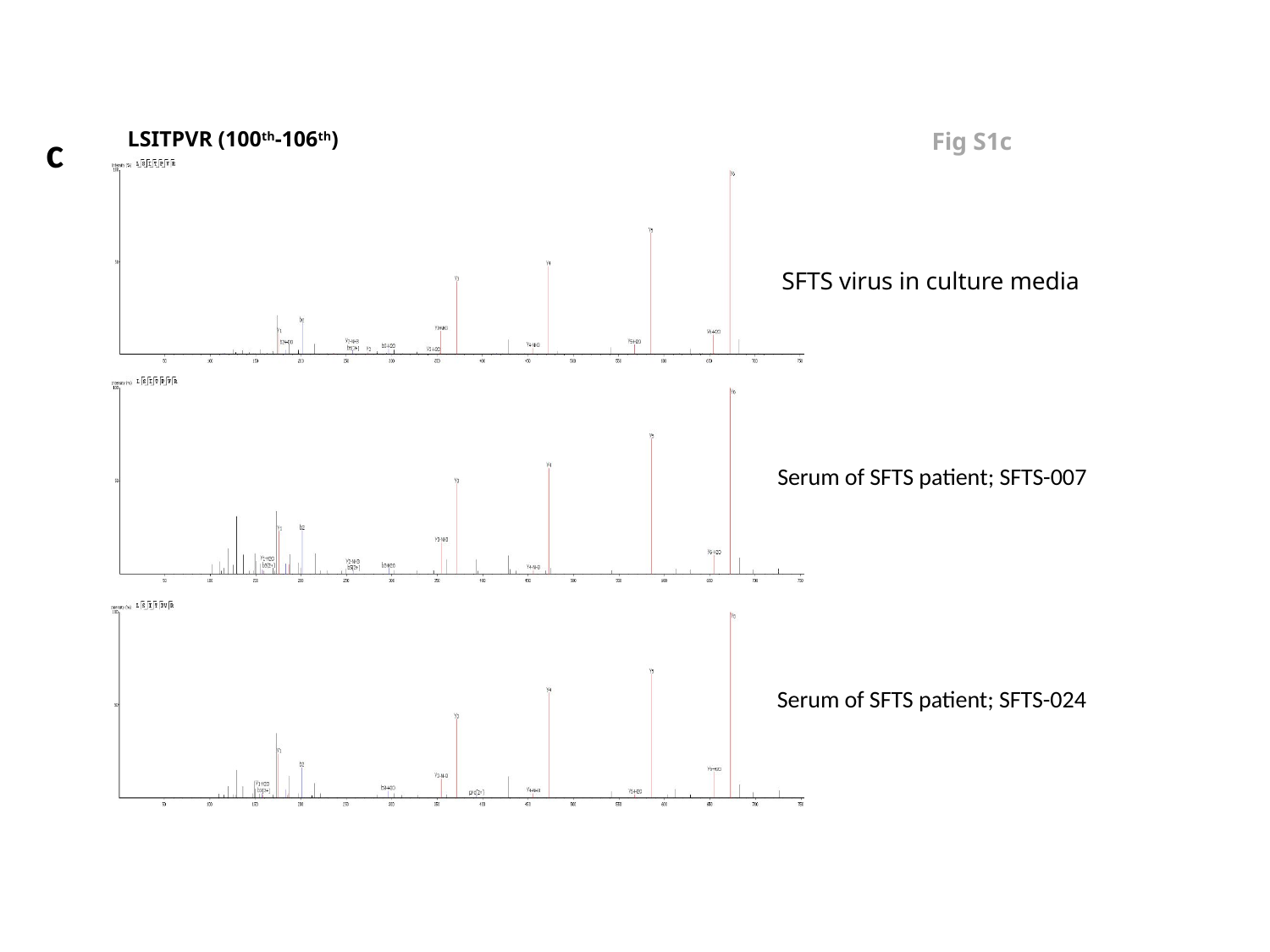

c
LSITPVR (100th-106th)
Fig S1c
SFTS virus in culture media
Serum of SFTS patient; SFTS-007
Serum of SFTS patient; SFTS-024

## Slide 5
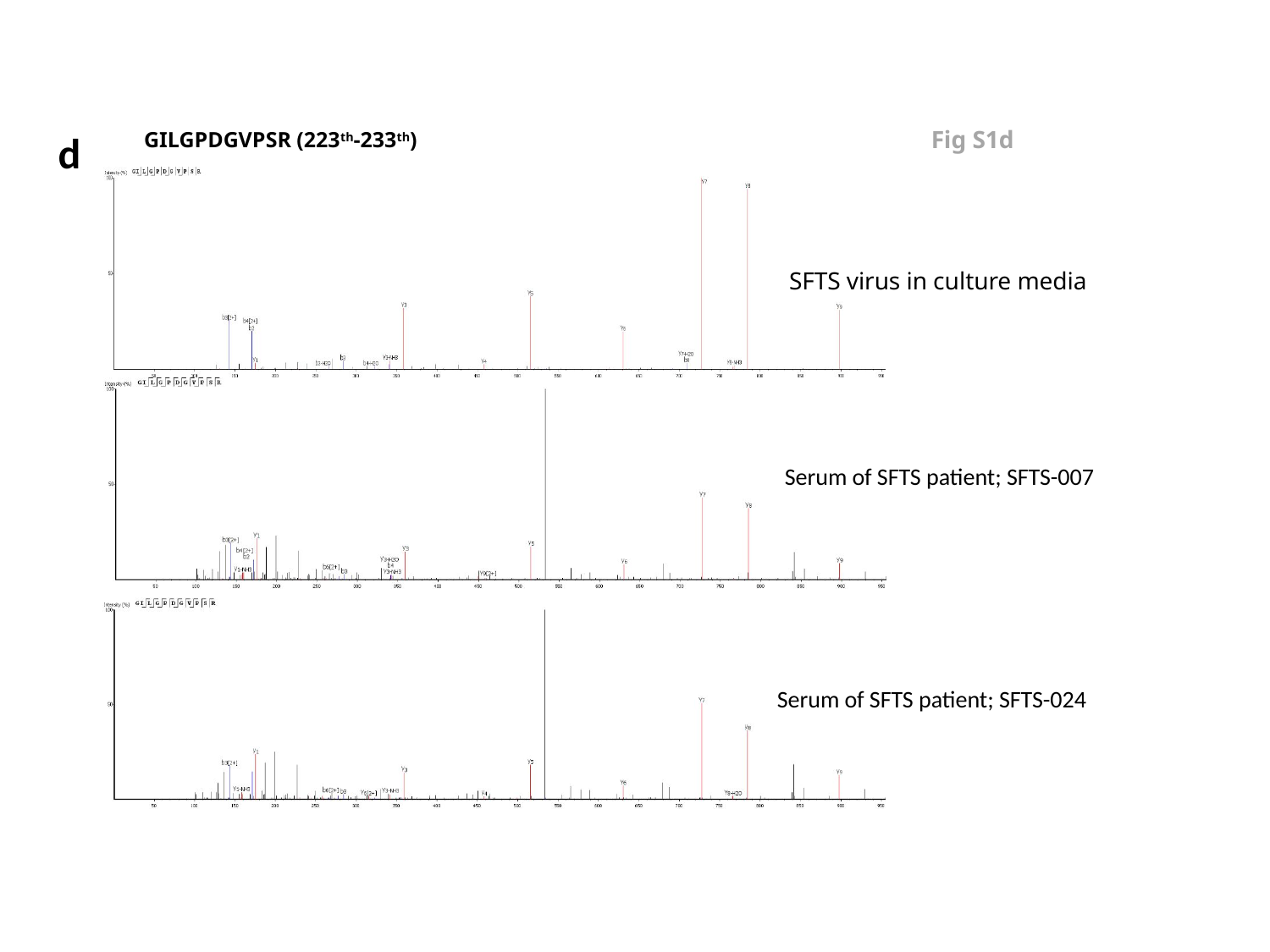

Fig S1d
d
GILGPDGVPSR (223th-233th)
SFTS virus in culture media
Serum of SFTS patient; SFTS-007
Serum of SFTS patient; SFTS-024
