# Supplementary material for: Proteomics-based diagnostic peptide discovery for severe fever with thrombocytopenia syndrome virus in patients
Source: Clin Proteomics. 2022 Jul 16;19:28. doi: 10.1186/s12014-022-09366-w (PMC9287713; doi:10.1186/s12014-022-09366-w)
Supplement: Supplementary file 3 — Additional file 3: Table S2. Target peptides and transitions used for the LC-PRM MS Analysis. [file 12014_2022_9366_MOESM3_ESM.docx]

**Additional File 3: Table S2. Target peptides and transitions used for the LC-PRM MS Analysis**

| Uniprot Accession | Protein Name | Peptide Modified Sequence | Precursor ion, m/z (charge) |
| --- | --- | --- | --- |
| [V5XK21](https://www.uniprot.org/uniprot/V5XK21) | Nucleocapsid protein | ELAYEGLDPALIIK | 772.9322 (+2, light) |
|  |  |  | 776.9393 (+2, heavy) |
|  |  | GILGPDGVPSR | 534.2958 (+2, light) |
|  |  |  | 539.3 (+2, heavy) |
