# Supplementary material for: Proteomics-based diagnostic peptide discovery for severe fever with thrombocytopenia syndrome virus in patients
Source: Clin Proteomics. 2022 Jul 16;19:28. doi: 10.1186/s12014-022-09366-w (PMC9287713; doi:10.1186/s12014-022-09366-w)
Supplement: Supplementary file 4 — Additional file 4: Figure S2. Qualitative characteristics of the PRM assay. The representative chromatograms of target peptides used in the PRM assay. a) Upper rows designated native peptides in serum specimens and b) lower rows designated heavy peptides of them. Mass error and retention times are annotated on the peak. c) Retention time of two target native peptides (light) and their synthetic peptides (heavy). The red bar denoted light peptides and blue bar denoted heavy peptides. [file 12014_2022_9366_MOESM4_ESM.pptx]

## Slide 1
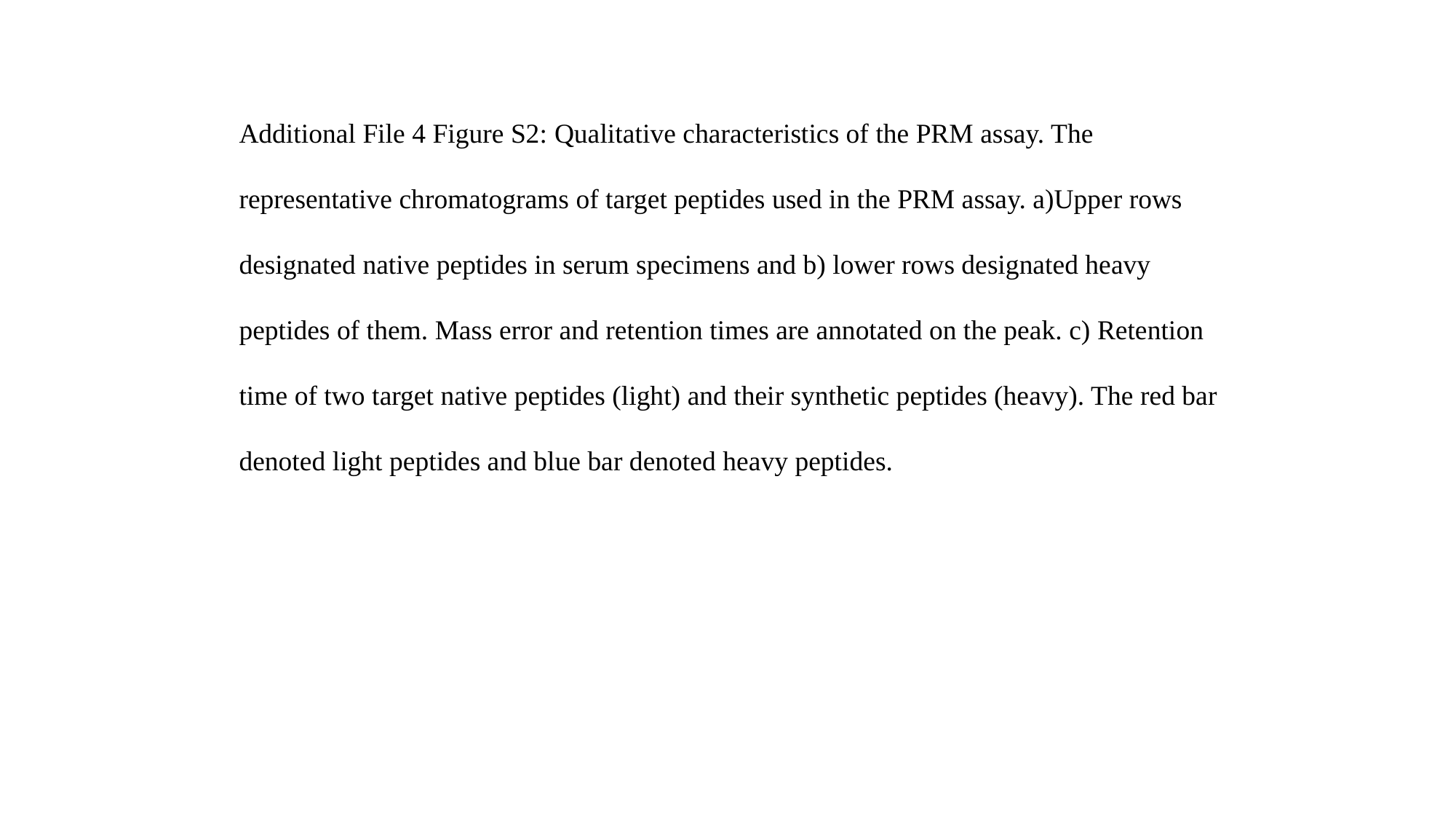

Additional File 4 Figure S2: Qualitative characteristics of the PRM assay. The representative chromatograms of target peptides used in the PRM assay. a)Upper rows designated native peptides in serum specimens and b) lower rows designated heavy peptides of them. Mass error and retention times are annotated on the peak. c) Retention time of two target native peptides (light) and their synthetic peptides (heavy). The red bar denoted light peptides and blue bar denoted heavy peptides.

## Slide 2
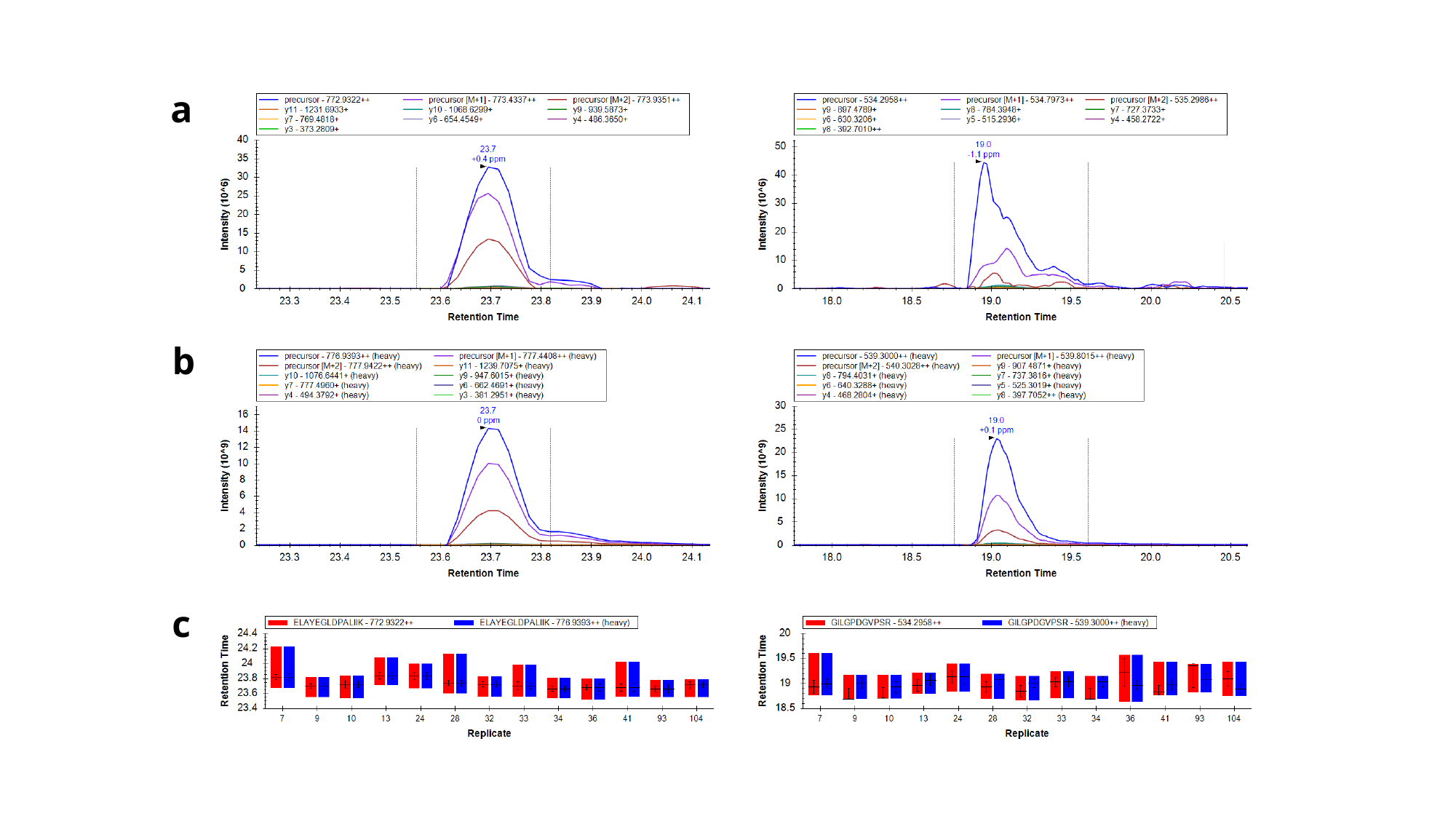

a
b
c
