# Supplementary material for: Proteomics-based diagnostic peptide discovery for severe fever with thrombocytopenia syndrome virus in patients
Source: Clin Proteomics. 2022 Jul 16;19:28. doi: 10.1186/s12014-022-09366-w (PMC9287713; doi:10.1186/s12014-022-09366-w)
Supplement: Supplementary file 5 — Additional file 5: Figure S3. 3D structure of the N protein and peptides detected by MS/MS analysis. Each peptide is assigned a different color: red (7–26th), orange (100–136th), yellow (165–179th), green (191–215th), or blue (223–233th). [file 12014_2022_9366_MOESM5_ESM.pptx]

## Slide 1
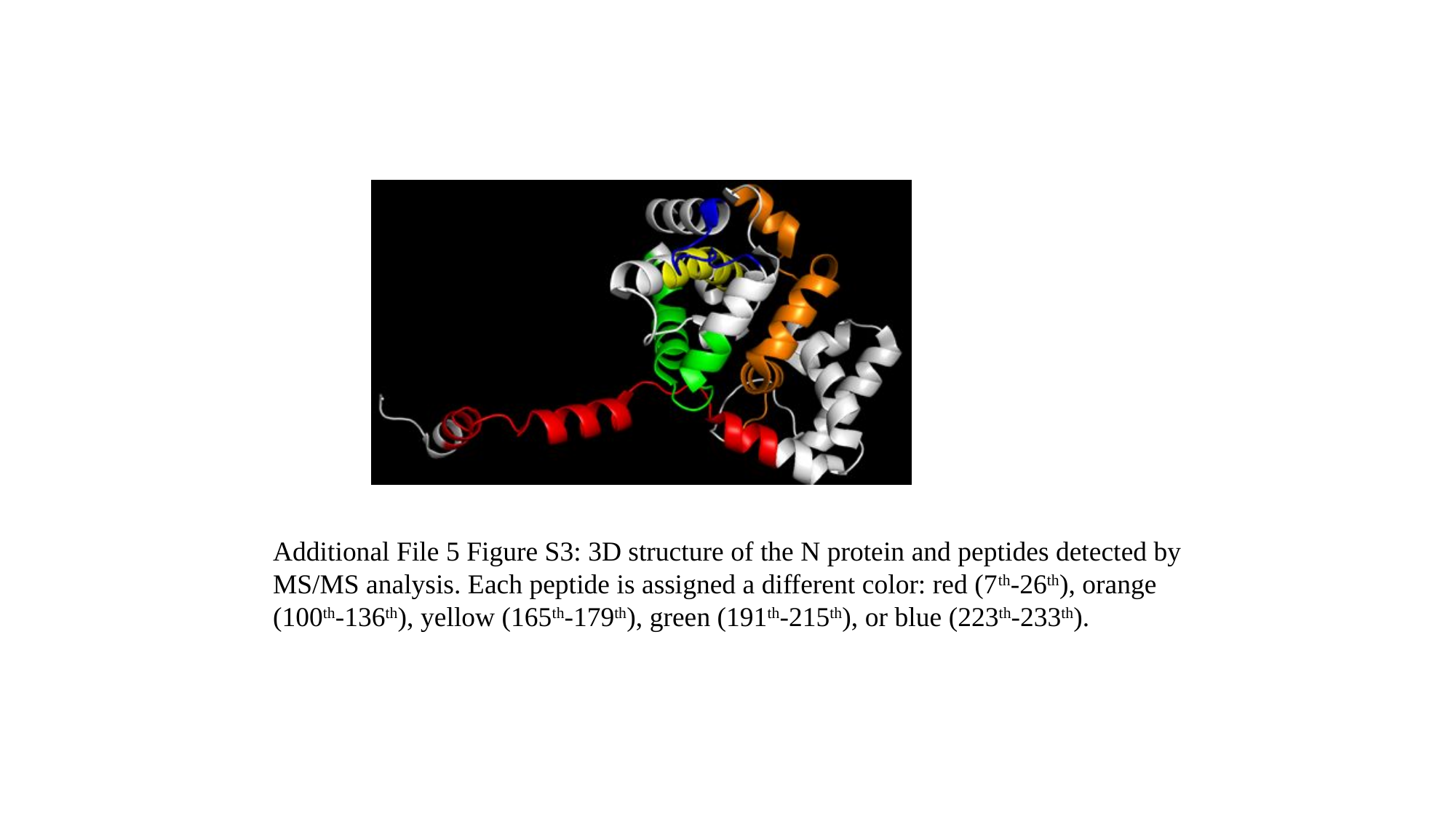

Additional File 5 Figure S3: 3D structure of the N protein and peptides detected by MS/MS analysis. Each peptide is assigned a different color: red (7th-26th), orange (100th-136th), yellow (165th-179th), green (191th-215th), or blue (223th-233th).
